# Evaluating the Performance of a Climate-Driven Mortality Model during Heat Waves and Cold Spells in Europe

**DOI:** 10.3390/ijerph120201279

**Published:** 2015-01-23

**Authors:** Rachel Lowe, Joan Ballester, James Creswick, Jean-Marie Robine, François R. Herrmann, Xavier Rodó

**Affiliations:** 1 Institut Català de Ciències del Clima (IC3), Carrer Doctor Trueta, 203, 3a, 08005 Barcelona, Spain; E-Mails: joan.ballester@ic3.cat (J.B.); xavier.rodo@ic3.cat (X.R.); 2 Division of Geological and Planetary Sciences (GPS), California Institute of Technology (Caltech), Pasadena, CA 91125, USA; 3 World Health Organization (WHO) Regional Office for Europe, European Centre for Environment and Health, Platz der Vereinten Nationen 1, 53113 Bonn, Germany; E-Mail: creswickj@who.int; 4 National Institute of Health and Medical Research, INSERM U988 and U1198, Université Montpellier II, U1198 MMDN—Bâtiment 24, Place Eugène Bataillon—CC105, 34095 Montpellier Cedex 05, France; E-Mail: jean-marie.robine@inserm.fr; 5 Ecole Pratique des Hautes Etudes (EPHE), Paris 75014, France; 6 Division of Geriatrics, Department of Internal Medicine, Rehabilitation and Geriatrics, Geneva University Hospitals, University of Geneva, Ch. Pont-Bochet, 1226 Thônex, Switzerland; E-Mail: francois.herrmann@hcuge.ch; 7 Institució Catalana de Recerca i Estudis Avançats (ICREA), Passeig de Lluís Companys, 23, 08010 Barcelona, Spain

**Keywords:** temperature, extremes, mortality, probabilistic, model, early warning, climate services

## Abstract

The impact of climate change on human health is a serious concern. In particular, changes in the frequency and intensity of heat waves and cold spells are of high relevance in terms of mortality and morbidity. This demonstrates the urgent need for reliable early-warning systems to help authorities prepare and respond to emergency situations. In this study, we evaluate the performance of a climate-driven mortality model to provide probabilistic predictions of exceeding emergency mortality thresholds for heat wave and cold spell scenarios. Daily mortality data corresponding to 187 NUTS2 regions across 16 countries in Europe were obtained from 1998–2003. Data were aggregated to 54 larger regions in Europe, defined according to similarities in population structure and climate. Location-specific average mortality rates, at given temperature intervals over the time period, were modelled to account for the increased mortality observed during both high and low temperature extremes and differing comfort temperatures between regions. Model parameters were estimated in a Bayesian framework, in order to generate probabilistic simulations of mortality across Europe for time periods of interest. For the heat wave scenario (1–15 August 2003), the model was successfully able to anticipate the occurrence or non-occurrence of mortality rates exceeding the emergency threshold (75th percentile of the mortality distribution) for 89% of the 54 regions, given a probability decision threshold of 70%. For the cold spell scenario (1–15 January 2003), mortality events in 69% of the regions were correctly anticipated with a probability decision threshold of 70%. By using a more conservative decision threshold of 30%, this proportion increased to 87%. Overall, the model performed better for the heat wave scenario. By replacing observed temperature data in the model with forecast temperature, from state-of-the-art European forecasting systems, probabilistic mortality predictions could potentially be made several months ahead of imminent heat waves and cold spells.

## 1. Introduction

An increase in the occurrence of extreme temperature events has been observed in Europe, especially during summer [1,2]. Temperature extremes are a threat to human health and are associated with a rise in mortality [3,4] among vulnerable segments of the population. The physiological basis for the adverse health effects of heat are well known and documented, as well as many social and environmental determinants of heat-related mortality and vulnerability factors. Exposure to high environmental temperatures can result in heat stress; mild to moderate heat-related health problems include heat rash, heat oedema, heat syncope, heat cramps and heat exhaustion [5]. Subsequent thermoregulatory failure and impaired regulation of inflammatory and stress responses facilitate the progression from heat stress to heat stroke and contribute to the severity of tissue injury [6]. The most vulnerable groups are the very elderly [7], and those with selective chronic illnesses [8,9], pre-existing conditions [10] or co-morbidities [11]. Low mean winter temperatures and exposure to cold weather are shown to be significantly associated with a variety of negative health outcomes, including high levels of cardiovascular-, respiratory-, cerebrovascular-, and all-cause morbidity and mortality [12,13,14], and specifically myocardial ischemia, myocardial infarction and sudden death [15,16,17]. Exposure to low temperatures can cause many physiological effects, including an increase in blood pressure, blood cholesterol, heart rate, plasma fibrinogen, platelet viscosity and peripheral vasoconstriction [18]. Vulnerable populations in cold winter weather are the elderly, rural and, generally, populations living in moderate winter climates [19]. A central European study has shown that cold stress has a considerable impact on mortality, comparable to the public health threat posed by heat waves [20].

Populations typically exhibit an optimum temperature with a corresponding lowest mortality rate; mortality rises at temperatures above or below this comfort zone. As such, seasonal patterns of mortality have been observed. Each episode of extreme temperature which lasts more than a couple of days can cause thousands of casualties as testified by the 2003 heat wave or the 2012 cold spell observed in western Europe. For example, over 70,000 excess deaths were observed in 12 European countries during the heat wave summer of 2003 [4].

Global average temperatures are projected to increase between 1.8 and 4.0 °C by end of this century, with a general increase overall and significant changes in regional temperature extremes [21,22,23]. The life of thousands of people may be threatened by a predicted increase in the frequency, length and intensity of temperature-extremes in the future [24,25]. Demographic changes expected this century (*i.e.*, increased life expectancy) mean that the health protection of elderly people will become a major challenge for health protection [26]. The degree at which death rates will increase will depend on the adaptive capacity of a population, including: acclimatization to higher temperatures; changes in the urban landscape to reduce heat-island effects; implementation of educational campaigns; and current health system preparedness and capacity building. A reduction of increased heat-related mortality can be achieved through reducing indoor heat exposure, implementation of heat-health action plans, and targeting particular care for vulnerable population groups [5]. It has been suggested that high winter mortality in Southern and Western Europe could be reduced through improved protection from the cold indoors and increased public spending on health care [27], as well as development and implementation of cold weather plans.

Heat–health action plans and cold weather plans depend on reliable early-warning systems to allow for long-term resource planning (e.g., energy and urban design), as well as timely activation of health action plans, to help local authorities prepare and respond to emergency situations. Interventions range from public awareness raising (information provided via the media giving behavioural and medical advice to the public), to specific information to actors within the health system. Health service delivery needs to be assured at all times, particularly when challenged during times of crisis, such as during summer heat wave emergencies. Climate forecasts would allow for better short-to-medium-term resource management within health systems, at the national level down to primary health care providers at local level, as well as allowing monitoring of the situation in neighbouring regions. Thus, the target group for this information is primarily health administration professionals and public health authorities, as well as all partners responsible for monitoring and implementing heat–health action plans and cold weather plans. Generally, countries, regions or cities having experienced a severe heat wave or cold spell in the past have short-term action plans ready to be executed. However, several countries, regions and cities do not yet have such plans in place [28]. For such cases, a warning several months before an extreme event could be very valuable. For countries that have not experienced a severe heat or cold event for several years, an early warning a few months before the event could allow time to update action plans.

Over the last few years, the quality of seasonal forecasts for average temperature conditions and precipitation totals have significantly improved, with our capacity to anticipate mean temperatures over land being higher in spring both in North America and in Europe [29,30]. The quality of seasonal climate forecasts increases during the development of El Niño events, particularly in the tropics. In the extratropics and particularly in Europe, seasonal climate forecasts currently have relatively limited forecast quality [31]. However, predicting extremes in both temperatures and precipitation has been possible in some areas (e.g., the Euro-Atlantic sector) by the incorporation of additional predictors into global climate models, such as snow cover and sea-ice. A new generation of climate prediction models are being developed to improve forecast quality in the near future [31]. These forecasts are currently being included in a regional framework of developing climate services in Europe [32].

The opportunity provided by the availability of meteorological data; and monthly-to-seasonal climate forecasts to create a valuable climate service relevant to the health sector has so far only been partially realised. As part of the EuroHEAT project, the German Weather Service developed an online medium-range heat information tool (http://euroheat-project.org/dwd/), which maps the probability of a forthcoming heat wave in the next 10 days using the ensemble prediction system of the European Centre for Medium-Range Weather Forecasts (ECMWF) [33,34]. This tool currently serves as the basis for monitoring heat wave probability for the WHO Regional Office for Europe. However; the tool is limited to 10-day lead times and is purely a meteorological tool; providing no indication of potential public health impact.

Decision-makers often require probabilistic information on which to base their decisions. However, communicating information contained within a probabilistic forecast can be a challenge. One way to summarise probabilistic information is to present the probability of exceeding predefined emergency thresholds. A decision to prepare for an event (e.g., an increase in summer mortality during a likely heat wave) might be taken when the forecast probability of surpassing the emergency threshold exceeds a predetermined “trigger” or decision threshold. In this study, we evaluate the performance of a climate-driven mortality model to provide probabilistic predictions of exceeding emergency mortality thresholds for heat wave and cold spell scenarios. The models are formulated using apparent temperature–mortality curves of average mortality at given temperatures for the period 1998–2003, for 54 socio-climatically distinct regions across Europe [3]. Using average mortality as the response variable and apparent temperature as the explanatory variable, a Bayesian model is formulated and posterior predictive distributions of mortality are simulated for specific climatic scenarios, based on daily apparent temperatures. According to the dataset (1998–2003), the warmest day across Europe occurred on 5 August 2003 and the coldest day on 8 January 2003. As a case study, we present probabilistic predictions of mortality across Europe during the 2003 summer heat wave and the 2003 winter cold spell. The advantage of simultaneously presenting mortality probability forecasts for different regions across Europe is that preparatory action can be directed to those regions with a greater likelihood of emergency mortality levels. As inputs to the model, we used observed (reanalysis) apparent temperature data as a “best guess” or upper limit for forecast temperatures. Ideally, “observed” temperature data would be replaced with an ensemble of temperature forecasts from state-of-the-art European forecasting systems. By using seasonal climate forecasts, probabilistic mortality predictions could potentially be made several months ahead of imminent heat waves and cold spells.

## 2. Experimental Section

### 2.1. Mortality, Population and Climate Data

Daily regional counts of mortality from 1 January 1998 to 31 December 2003 were collected for 187 NUTS2 regions (*i.e.*, second level of the Nomenclature of Territorial Units for Statistics) in 16 European countries; namely Austria, Belgium, Croatia, the Czech Republic, Denmark, France, Germany, Italy, Luxembourg, Netherlands, Poland, Portugal, Slovenia, Spain, Switzerland and the United Kingdom (England and Wales only) [4]. Population estimates at the regional level were provided by Eurostat. The mean population of the 187 NUTS2 regions was 2.1 million, with a range of 53,000 to 17.4 million. Due to the extremely large differences in population between regions, they were grouped in 54 larger and more homogeneously populated aggregations [3]. Daily high-resolution data (0.75° × 0.75°) of daily mean (Tair) and dew point (Tdewpt) temperature were derived from the reanalysis ERA-Interim dataset [35]. ERA-Interim is a global atmospheric reanalysis available from 1979 and continuously updated in real time. This gridded reanalysis data was interpolated to the regional level by taking into account the higher population density in cities and metropolitan areas. Daily mean apparent temperature was calculated according to Tapp = −2.653 + 0.994·(Tair) + 0.0153·(Tdewpt)^2^, with all variables expressed in °C [36,37,38,39]. Reanalysis data was used as a “best guest” for forecast temperatures.

### 2.2. Estimation of the Temperature-Mortality Relationship

The relationship between temperature and mortality has been traditionally described as a U-, V- or J-shaped correspondence [40], defined by a temperature range of minimum mortality and monotonically increasing incidence for colder and warmer temperatures [41]. A wide range of techniques are used to characterize this relationship, such as linear models [42], generalized linear models [36], non-linear regression models [43], non-parametric smoothing functions [44] or exposure-response epidemiological models [45]. The approach used in this study is based upon the methodology outlined in Ballester *et al.* [3], where apparent temperature and daily mortality transfer functions were formulated for 54 regions across Europe. Here, we extend this approach by estimating mortality—apparent temperature relationships in a Bayesian model framework, which allows the simulation of probabilistic predictions of daily mortality in space and time.

The temperature-mortality dependency for each aggregation was estimated as follows: The range of observed temperatures was divided in equally spaced intervals [46,47]. Days belonging to each interval were grouped and daily temperature and mortality data within each interval were averaged. Interval mean mortality was smoothed by means of a centred 31-term filter, corresponding to nearly 3 °C intervals [42]. The lowest value defines the interval of comfort temperature [41,48]. This threshold divides the range of temperatures into “warm” and “cold” tails. The model used to fit the temperature-mortality curves was formulated as follows:

$$y_{ik}\sim N\left(\alpha_{j}+\beta_{1j}x_{ik}+\beta_{2j}x_{ik}^{2}+\beta_{3j}x_{ik}^{3},\sigma_{j}^{2}\right),$$

where *y_ik_* is the logarithm of the average mortality rate (per million population) at region, *i* and temperature interval, *k*. Then, for each region *i*, the log mortality rate was formulated as a non-linear function of temperature, *x_ik_*, (a third order polynomial), with location specific intercept, α*_j_*. Note that parameters are fitted separately for the warm tail (*j* = *w*) and cold tail (*j* = *c*), depending on whether the temperature was greater than (*x_ik_* ≥ *x_im_*) or less than (*x_ik_* < *x_im_*) the comfort temperature (*i.e.*, the temperature of minimum mortality), *x_im_*. In other words, the so called “cold tail” and “warm tail” of the typical U, V or J shaped temperature–mortality curves are fitted separately. The comfort temperature typically occurs in Europe twice per year, around June and September. Therefore, the model controls for typically warmer (e.g., June–September) and colder months (October–May), but is flexible enough to allow for temperature extremes that occur outside of these seasons.

The models were fitted in a Bayesian probabilistic framework, using Integrated Nested Laplace Approximation (INLA, www.r-inla.org) [49,50]. INLA is a promising alternative to Markov Chain Monte Carlo (MCMC) methods, due to much shorter computational times. This is an advantage in a climate service framework, where models need to be regularly updated with the latest climate information, to support rapid decisions.

In order to simulate mortality predictions for heat wave or cold spell scenarios, spatio-temporal apparent temperature data, *x_it_*, where t is the time step (daily), for heat wave and cold spell scenarios were combined with 1000 samples of the parameters estimated from the warm tail model (*j* = *w*):

$$y_{it}\sim N\left(\alpha_{w}+\beta_{1w}x_{it}+\beta_{2w}x_{it}^{2}+\beta_{3w}x_{it}^{3},\sigma_{w}^{2}\right)$$

if *x_it_* ≥ *x_im_*

and cold tail model (*j* = *c*):

$$y_{it}\sim N\left(\alpha_{c}+\beta_{1c}x_{it}+\beta_{2c}x_{it}^{2}+\beta_{3c}x_{it}^{3},\sigma_{c}^{2}\right),$$

if *x_it_* < *x_im_*, respectively.

Therefore, 1000 samples of daily mortality rates were generated for each region and each day, *y_it_*. Daily mortality rates were then averaged for the climatological events of interest, *i.e.*, the heat wave period, 1–15 August 2003 and the cold spell period, 1–15 January 2003.

Region specific *emergency* thresholds of mortality rates were set at the 75th percentile (3rd quartile) of the mortality distribution, given temperatures greater than or less than the comfort temperature. This emergency threshold was selected as an initial first test, as it is commonly adopted as an emergency/detection threshold for other health outcomes [51]. Two probability *decision* thresholds were tested to evaluate the model; a “cautious” decision threshold of 30% and a more strict decision threshold of 70%. Previous case studies for 2006 have shown that under- and over-forecasting of heat events becomes minimal for an exceedance probability of 30% [34]. However, preliminary results from the engagement of users and stakeholders within the EUPORIAS project have provided anecdotal evidence that some decision-makers tend towards higher certainty thresholds, with a 70% threshold proposed by some users [52]. By using a probability decision “cut off”, the efficacy of the model for these specific scenarios was evaluated in a binary framework. To assess the correspondence between forecasts and observations, the proportion of correct predictions, and conditional probabilities such as the hit rate and the false alarm rate were calculated. For this exercise, the “proportion correct” is defined as the proportion of the 54 regions for which the model correctly anticipated that mortality rates would or would not exceed the emergency threshold. The “hit rate” (sensitivity) is the proportion of regions that correctly predicted that the emergency threshold would be exceeded. Conversely, the false alarm rate (1-specificity) is the proportion of regions for which the mortality rate was predicted to exceed the emergency threshold, but did not.

For any event, a graph can be constructed (known as a relative operating characteristic (ROC) curve) that indicates the hit rates and false alarm rates that would result from using different probability decision thresholds [53]. The ROC score (equivalent to the area under the modelled ROC curve), is a widely used measure of skill. The ROC score characterises the quality of a forecast system by describing the system’s ability to anticipate correctly the occurrence or non-occurrence of pre-defined events. A ROC score value of 50% indicates zero skill while a value of 100% represents perfect skill.

## 3. Results and Discussion

### 3.1. Temperature and Mortality Curves

**Figure 1:**
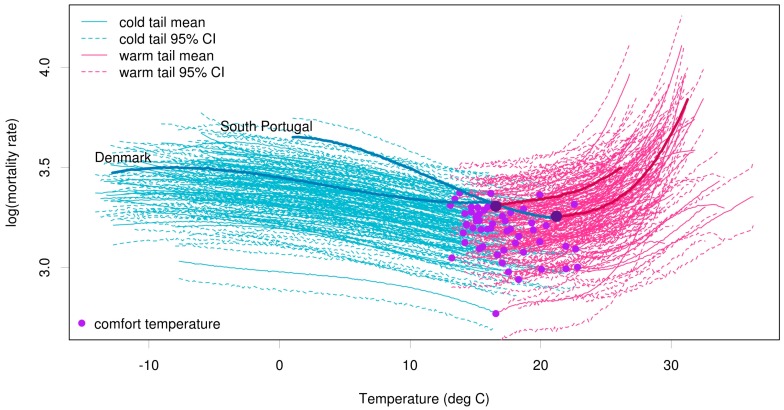
Posterior predictive distributions (mean and 95% credible intervals) for cold tail (blue) and warm tail (pink) estimations for all 54 regions across Europe. The comfort temperature threshold for each region is marked with a purple dot. The mean mortality curves for two contrasting regions (South Portugal and Denmark) are magnified.

Figure 1 shows the mean and 95% credible intervals (Bayesian equivalent of confidence intervals) obtained from the probabilistic simulations of the Bayesian probabilistic model, for each of the 54 regions. The region-specific comfort temperature, *i.e.*, the temperature at minimum mortality, is indicated by a purple dot. This comfort temperature occurs twice a year around June and September, defining a summer season of warm tail temperatures with non-linear mortality sensitivity and a long 3-season period (autumn, winter and spring) of cold tail temperatures with near-linear mortality increases with decreasing temperature [3].

To demonstrate the difference in comfort temperature and the rate at which mortality increases due to heat or cold, the mortality curves for two contrasting regions, namely South Portugal and Denmark, are highlighted. The comfort temperature is higher in South Portugal than in Denmark. Portugal is more sensitive to temperature changes than Denmark, with increases in mortality in both the warm and cold tails occurring at a much greater rate.

### 3.2. Probability of Exceeding Emergency Mortality Thresholds

**Figure 2:**
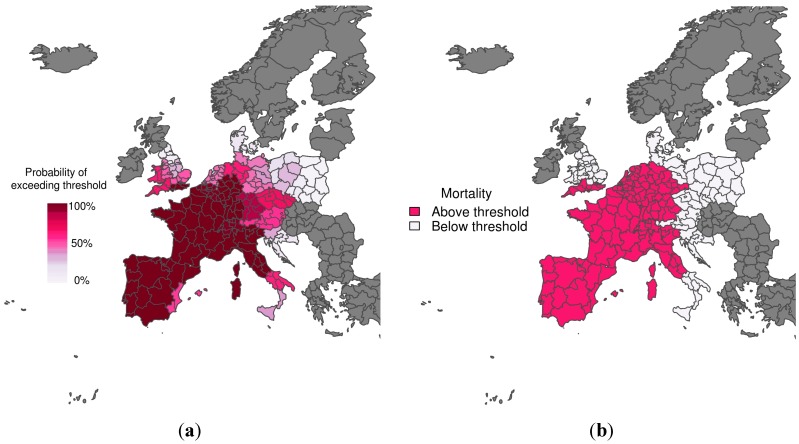
(**a**) Probabilistic map of exceeding emergency daily mortality thresholds (75th percentile of daily mortality distribution in the warm tail); (**b**) Corresponding observations during a heat wave scenario (1–15 August 2003). The graduated colour bar represents the probability of exceeding the mortality threshold (ranging from 0%, pale colours, to 100%, deep colours).

Figure 2 shows the predicted probability of mortality rates exceeding the 75th percentile of the mortality distribution, given that temperatures are greater than the comfort temperature (*i.e.*, the warm tail distribution) for the heat wave period 1–15 August 2003. The corresponding observations (*i.e.*, whether the mortality rate exceeded the threshold or not) are also displayed. The colour used to represent the observed mortality rates above the emergency threshold (Figure 2b) corresponds to the colour used to represent a 70% probability of exceeding the emergency threshold (Figure 2a). The predictions and observations agree quite well, with the model correctly predicting with high confidence that mortality would exceed the emergency threshold across most of Spain, France and Northern Italy. There was a much lower probability of exceeding the emergency threshold in Southern Italy and North East England. When compared with observations, these areas represent correct rejections when using a decision threshold of 70%.

**Figure 3:**
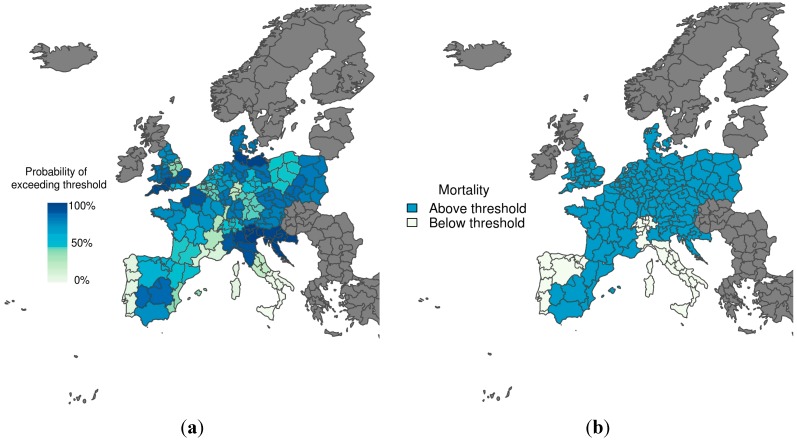
(**a**) Probabilistic map of exceeding emergency daily mortality thresholds (75th percentile of daily mortality distribution in the cold tail); (**b**) Corresponding observations during a cold wave scenario (1–15 January 2003). The graduated colour bar represents the probability of exceeding the mortality threshold (ranging from 0%, pale colours, to 100%, deep colours).

Figure 3 shows the predicted probability of mortality rates exceeding the 75th percentile of the mortality distribution, given that temperatures are colder than the comfort temperature (*i.e.*, the cold tail distribution) for the winter period 1–15 January 2003. The corresponding observations (*i.e.*, whether the mortality rate exceeded the threshold or not) are also displayed, with the colour used to represent observed mortality rates above the emergency threshold (Figure 3b) corresponding to the colour used to represent a 70% probability of exceeding the emergency threshold (Figure 3a). The model does a reasonable job at predicting mortality rates above the emergency threshold, with high certainty correctly predicted in Croatia and South West England, for example. The model also correctly predicted a very low chance of exceeding the mortality threshold in Portugal and Central and South Italy. However, false alarms would have resulted for northwestern Italy, using both the more conservative (30%) and more strict (70%) probability trigger threshold.

### 3.3. Assessment of Probability Decision Thresholds

Table 1 shows an evaluation of the probabilistic predictions for the heat wave and cold spell scenarios examined in this study. As describe above, probability decision thresholds of 70% and 30% were selected a priori. Given the pre-defined emergency and probability decision thresholds, using the average of daily temperatures across Europe from 1–15 August 2003 (summer heat wave) would have resulted in a hit rate of 85%, a false alarm rate of 5% and an overall proportion of correct predictions of 89% when using the stricter decision threshold of 70%. When using the more cautious decision threshold of 30%, no instances of exceeding the emergency threshold are missed, resulting in a 100% hit rate, but the false alarm rate increases substantially to 55% and the proportion correct decreases. For the cold spell scenario, the model performed less well, with a hit rate of 66%, a false alarm rate of 20% and proportion of correct predictions of 69% when using a decision threshold of 70%. Lowering the decision threshold to 30% increases the false alarm rate, although to a lesser extent than for the heat wave scenario. The overall proportion correct increases, suggesting that a more conservative probability decision threshold might be more suitable for predicting cold-related mortality. Overall, the model performed best for the heat wave scenario, with a ROC score of 97%. The ROC score for the cold spell scenario was 78%.

**Table 1:** Evaluation of heat waves and cold spell scenarios given pre-defined emergency and probability decision thresholds.

Scenario	Emergency Threshold Defined for Each Region Using Daily Data 1998–2003	ROC Score	Probability Decision Threshold	Hit Rate	False Alarm Rate	Proportion Correct
Heat wave 1–15 August 2003	75th percentile of mortality distribution given that temperature is warmer than the comfort temperature.	97%	70%	85%	5%	89%
			30%	100%	55%	80%
Cold spell 1–15 January 2003	75th percentile of mortality distribution given that temperature is colder than the comfort temperature.	78%	70%	66%	20%	69%
			30%	93%	40%	87%

## 4. Discussion and Conclusions

The results from this analysis demonstrate that the model was successfully able to anticipate the occurrence or non-occurrence of mortality rates exceeding the emergency threshold (75th percentile of the mortality distribution) in most regions, for both heat wave and cold spell scenarios. The overall performance of the model was better for the heat wave scenario. The relationship between temperature and mortality for 54 large regions spanning Europe was investigated in this study in order to find the time lag that maximizes the amount of explained variance [54]. In general, this fraction is maximum in the warm tail at very short time lags (*i.e.*, 0–1 days), with a sharp decrease for immediately longer lags (e.g., [48,55]). The physiological effects of cold weather on human health are complex and are thus more difficult to capture in a daily temperature surveillance system. Studies in China have shown that the health effects of cold were delayed whereas the effects of high temperatures where either immediate or with only a short time lag [13]. In this study, the optimal lag in the cold tail was found to be at one week, with a plateau in explained variance for shorter lags and decreasing values for longer lags (see also [44,48]). Given that the model is designed to estimate the relationship between temperature and mortality for every day of the year, across a large geographical area, the same lag was imposed for the warm and cold tails, so that each day is unequivocally assigned to one of the two tails [3]. For simplicity, and taking into account the lag-dependency in the amount of explained variance, the zero lag was chosen. This lag is shown to be valid for the events considered in the present work, given that mortality incidence was found to increase on average 0–1 days after the summer 2003 heat wave and up to a week after extreme temperatures during the winter 2003 cold spell.

Despite this, non-thermal seasonal factors affecting mortality are not taken into account in the model, such as air pollution and infectious diseases. An observational study in southeast England from 1990–2000 showed that only 2.4% of excess winter deaths were either directly or indirectly due to influenza [56]. According to Keatinge [57], approximately half of excess winter deaths were due to coronary thrombosis (due to cold exposure, peaking about two days after the peak cold), and approximately half the remaining winter deaths were caused by respiratory disease (peaking about 12 days after peak cold).

Air pollution and social deprivation [58] have been shown to act in synergy with temperature changes, during both the cold [59] and the warm seasons, to increase mortality [60,61]. Studies have shown that the compounding effects of pollution on temperature exposure can result in delayed respiratory mortality over several weeks [62]. Thus, a short time lag insufficiently captures all the effects of temperature-related cerebrovascular mortality and longer time lags would be required. However, without specific information about the peculiarities of each regions, which could result in differential lags across Europe, assuming the same lag in every case for each region for prediction purposes facilitates the future near-real time implementation of a generalized and automated early warning system, incorporating climate forecast information.

Although the spatial coverage of the mortality dataset is rich, this analysis was limited by the relatively short time period for which the data was available (1998–2003). The data collection is currently being expanded to cover the years 1998–2012, and mortality rates at the NUTS-2 level will be computed using daily population estimates calculated according to an improvement (age range 0–100) to the original method described previously (which was limited to age range 63–age maximum) [7]. In light of these initial findings, the research will evolve in two stages: Firstly, using the updated detailed database, the relationship between temperature and mortality will be revised for each region, to fully investigate the risk for different vulnerability groups, taking into account time lags (particularly for cold extremes) and allowing for modifying factors, such as differential health care provision, insulation and air conditioning practices between regions. Model accuracy might be improved by using explanatory variables such as air pollution and sentinel respiratory infection data. However, unlike climate variables, seasonal forecasts of such confounding factors are not readily available.

Secondly, the updated model framework will be used to assess the viability of incorporating forecast (instead of reanalysis) temperatures. In order to account for the additional source of uncertainty generated by using forecast instead of observed temperatures, an ensemble of temperature forecasts will be incorporated into the probabilistic Bayesian framework. These forecasts will be obtained from the European Climate Observations, Modelling and Services initiative (ECOMS) User Data Gateway [63]. Increased forecast lead-time is usually at the cost of a reduction in spatial and temporal resolution of the predicted outcome. The trade-off between predictive lead-time (up to many months ahead) and mortality prediction uncertainty will be examined, to determine optimum lead times and spatial/temporal resolution for mortality predictions across Europe.

Health has been identified as a priority of the Global Framework for Climate Services (GFCS) [64] with a mandate to better support and inform the health sector of climate risks, by increasing the capacity of health sector to effectively access, understand and use climate and weather information for health decisions and to mainstream climate and weather data to health operations [65]. Through an iterative evaluation process with public health decision-makers, we plan to develop this work into a key prototype climate service for public health. A technical meeting is planned for 2015 to bring together the model developers and data providers with members of the international and national health communities and decision-makers to further align development with the needs and understanding of future users.
